# Anti-CXCL4 Antibody Reactivity Is Present in Systemic Sclerosis (SSc) and Correlates with the SSc Type I Interferon Signature

**DOI:** 10.3390/ijms21145102

**Published:** 2020-07-19

**Authors:** Roberto Lande, Anna Mennella, Raffaella Palazzo, Immacolata Pietraforte, Katia Stefanantoni, Nicoletta Iannace, Alessia Butera, Monica Boirivant, Roberta Pica, Curdin Conrad, Carlo Chizzolini, Valeria Riccieri, Loredana Frasca

**Affiliations:** 1 Istituto Superiore di Sanita’, National Centre for Pre-Clinical and Clinical Drug Research and Evaluation, Pharmacological Research and Experimental Therapy Unit, 00166 Rome, Italy; roberto.lande@iss.it (R.L.); anna.mennella@guest.iss.it (A.M.); raffaella.palazzo@iss.it (R.P.); alessia.butera@iss.it (A.B.); monica.boirivant@iss.it (M.B.); 2 Department of Dermatology, University Hospital CHUV, 1011 Lausanne, Switzerland; curdin.conrad@chuv.ch; 3 Department of Oncology and Molecular Medicine, Istituto Superiore di Sanità, 00161 Rome, Italy; immacolata.pietraforte@iss.it; 4 Division of Rheumatology, Internal Medicine and Medical Specialties, University La Sapienza, 00185 Rome, Italy; katia.stefanantoni81@gmail.com (K.S.); nicoletta_iannace@live.it (N.I.); valeria.riccieri@uniroma1.it (V.R.); 5 Sandro Pertini Hospital, IBD, GE Unit, 00157 Rome, Italy; roberta.pica@aslroma2.it; 6 Immunology & Allergy and Immunology & Pathology Dept., University Hospital and School of Medicine, CH-1211 Geneva, Switzerland; carlo.chizzolini@unige.ch

**Keywords:** autoantibodies, CXCL4, type I interferon, lung fibrosis, innate immunity, adaptive immunity

## Abstract

Systemic sclerosis (SSc) is characterized by skin/internal organ fibrosis, vasculopathy and autoimmunity. Chemokine (C-X-C motif) ligand 4 (CXCL4) is an SSc biomarker, predicting unfavorable prognosis and lung fibrosis. CXCL4 binds DNA/RNA and favors interferon (IFN)-α production by plasmacytoid dendritic cells (pDCs), contributing to the type I IFN (IFN-I) signature in SSc patients. However, whether CXCL4 is an autoantigen in SSc is unknown. Here, we show that at least half of SSc patients show consistent antibody reactivity to CXCL4. T-cell proliferation to CXCL4, tested in a limited number of patients, correlates with anti-CXCL4 antibody reactivity. Antibodies to CXCL4 mostly correlate with circulating IFN-α levels and are significantly higher in patients with lung fibrosis in two independent SSc cohorts. Antibodies to CXCL4 implement the CXCL4–DNA complex’s effect on IFN-α production by pDCs; CXCL4–DNA/RNA complexes stimulate purified human B-cells to become antibody-secreting plasma cells in vitro. These data indicate that CXCL4 is indeed an autoantigen in SSc and suggest that CXCL4, and CXCL4-specific autoantibodies, can fuel a harmful loop: CXCL4–DNA/RNA complexes induce IFN-α in pDCs and direct B-cell stimulation, including the secretion of anti-CXCL4 antibodies. Anti-CXCL4 antibodies may further increase pDC stimulation and IFN-α release in vivo, creating a vicious cycle which sustains the SSc IFN-I signature and general inflammation.

## 1. Introduction

Systemic sclerosis (SSc) is an autoimmune disease whose hallmarks are autoimmunity, fibrosis and vasculopathy [1]. Autoimmunity is an important component, as autoreactive T-cells and autoantibodies play a central role in SSc pathogenesis [1,2,3,4]. According to the extension of skin fibrosis, we can distinguish two major SSc subsets: limited cutaneous (lcSSc) and diffuse cutaneous (dcSSc) [5]. Dysregulation of the innate immune system in genetically predisposed individuals seems to be the leading cause of SSc, and aberrant Toll-like receptor (TLR) activation appears central to the pathogenesis [3,6].

A recent proteomic study identified chemokine (C–X–C motif) ligand 4 (CXCL4) (also known as platelet factor 4, PF4) as a biomarker of SSc, particularly in early active diffuse SSc [7]. CXCL4-releasing SSc-derived plasmacytoid dendritic cells (pDCs), a cell type crucially involved in the type I interferon (IFN-I) response, were shown to overproduce IFN-α when stimulated with synthetic oligonucleotide CpG [7,8]. These studies have provided the first link between CXCL4 overexpression and a type I interferon signature. Of note, an IFN-I signature at early onset seems linked to a poor SSc prognosis [9,10,11].

The interaction of innate and adaptive immune cells appears crucial in SSc. There are some examples of typical SSc autoantibodies that activate innate immune cells, fuel inflammation and can contribute to the IFN-I signature and fibrosis [9,10]. For instance, Kim’s group demonstrated that SSc sera containing autoantibodies that mark SSc, such as anti-centromere (ACA) and anti-topoisomerase (ATA) antibodies, induced high levels of IFN-α in healthy donor (HD) peripheral blood mononuclear cells (PBMCs) in a pDC- and RNA/DNA-dependent manner [9]. A paper by Eloranta et al. also showed that sera from SSc patients, mixed with necrotic/apoptotic material, induced IFN-α production in pDCs, apparently activated by immunoglobulin (Ig) immune complexes (ICs) formed by SSc autoantibodies [10]. Interestingly, SSc IgG immune complexes activated nucleic acid responsive TLRs (TLR7/8/9), suggesting that extracellular release of nucleic acids and endosomal TLRs are important in SSc pathogenesis [9,10].

In a recent paper, we found that stimulation of HD pDCs with diluted SSc plasma treated with DNA elicited IFN-α release by pDCs [12]. Of note, this secretion was significantly inhibited by the addition of an anti-constant fragment (Fc) receptor antibody, indicating that antibodies were at least partially responsible for the IFN-α production. Western blot analysis of IgG that were immunoprecipitated from SSc plasma revealed that IgG had some DNA attached [12]. We assumed that typical SSc antibodies could be present (such as ATA or ACA), but also that other autoantibodies with unknown specificity were likely contained in the blood mixture and maybe be attached to nucleic acids as well, and lead to inflammation.

In the same study, an additive and significant effect of DNA-treated plasma could be attributed to natural CXCL4 contained in blood, as an inhibitory anti-CXCL4 antibody blocked IFN-α production by pDCs in these experiments. We indeed demonstrated that CXCL4 enables innate immune recognition of natural DNA because it forms liquid crystalline complexes with both human DNA (self-DNA, huDNA) and microbial DNA (bacterial DNA, bacDNA) and also with RNA [12]. CXCL4 protects the DNA form degradation and allows DNA entry into the cells, while organizing it into periodic liquid crystalline structures with an inter-DNA spacing well matched with the steric size of TLR9. This enables strong activation of TLR9 in pDCs [12]. Most intriguingly, we demonstrated that CXCL4–DNA complexes exist in the circulation and tissue of SSc patients and correlate with IFN-α measured by ELISA, or with interferon responsive genes activation (Mx1), in blood and tissues (skin), respectively [12].

The ability of CXCL4 to bind cationic molecules including DNA, in the form of crystals, suggested to us that anti-CXCL4 antibodies could be easily generated in SSc. Indeed, formation of particulate structures, such as large molecular complexes and/or crystal structures containing self-molecules, confers greater antigenicity to otherwise less immunogenic proteins [13,14].

The data reveals that anti-CXCL4 antibody autoreactivity is present in a consistent proportion of SSc patients and correlates with IFN-α, while T-cells also recognized CXCL4 as an autoantigen, suggesting T-cell help for autoantibody production.

## 2. Results

### 2.1. SSc Patients Show Consistent Antibody Reactivity to CXCL4

**Figure 1:**
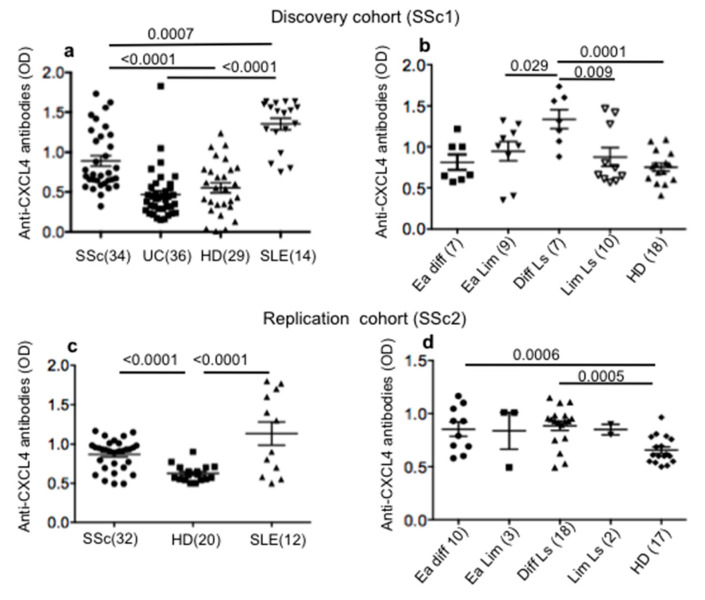
Anti-CXCL4 antibody reactivity is present in SSc patients. Sera (SSc1) (**a**,**b**) and plasma (**c**,**d**) (SSc2) and sera/plasma (see Table S1) of HD (healthy donors), UC (ulcerative colitis) and SLE (systemic lupus erythematosus) patients were tested for antibody reactivity to CXCL4 by ELISA as described in the Methods section. HD sera (**a**,**b**) or plasma (**c**,**d**) were used as negative controls, SLE sera (**a**) and SLE plasma (**c**) were used as positive controls. Positivity in patients was calculated as reported in the Methods section with respect to HD. In all graphs, horizontal bars are the mean, vertical bars are the standard error of the mean, *p* values are by Mann–Whitney test. Ea diff = early diffuse SSc; Ea Lim = early limited SSc; Diff Ls = diffuse long-standing SSc patients; Lim Ls = limited long-standing SSc patients (for early patients, disease duration is <5 years).

To address whether CXCL4 could be an autoantigen in SSc patients, we firstly tested antibody reactivity to CXCL4. We used the homemade ELISA described in the Methods section. As a control, we measured anti-CXCL4 antibody reactivity in HD. Since CXCL4 elevation is described in inflammatory bowel diseases [15], we addressed anti-CXCL4 reactivity in ulcerative colitis (UC) patients. Finally, we analyzed patients with systemic lupus erythematosus (SLE), as a study showed that antibodies specific for CXCL4 are present in SLE and correlate with disease activity [16]. The results in Figure 1a, relative to our discovery cohort (SSc1, see Table S1), show that 18 out of 34 SSc patients (53%) were positive for antibody reactivity to CXCL4, as compared to HD. The difference between HD and SSc was significant. In contrast, UC patients did not significantly respond to CXCL4. As expected, SLE patients (SLE1 cohort, see Table S1) harbored antibodies against CXCL4 [16]. As shown in Figure 1b, diffuse long-lasting patients belong to the group that mainly respond to CXCL4. One patient showed anti-CXCL4 antibody reactivity in the early diffuse group (14%) and three out of nine (33%) were responsive in the early limited group. Of note, in the same cohort, CXCL4 antibody reactivity correlated positively with disease duration (*r* = 0.34; *p* = 0.028; *n* = 32).

In the replication cohort (SSc2, see Table S1), 10 out 32 SSc patients (31%) showed autoantibody reactivity to CXCL4, which was significantly higher as compared to HD (Figure 1c). Even in this case, responses from SLE patients (SLE2, see Table S1) were present. Again, diffuse long-standing patients showed a significant antibody response to CXCL4 in the SSc2 cohort, but early diffuse patients also did in this cohort (Figure 1d).

Thus, these data show that antibody reactivity to CXCL4 characterizes SSc.

### 2.2. Antibody Reactivity to CXCL4 Correlates with Disease Score and Modified Rodnan Skin Score (mRSS) in Patients with Active Disease

Given the consistent reactivity of SSc antibodies to CXCL4, we wondered whether this antibody response correlated with disease activity.

**Figure 2:**
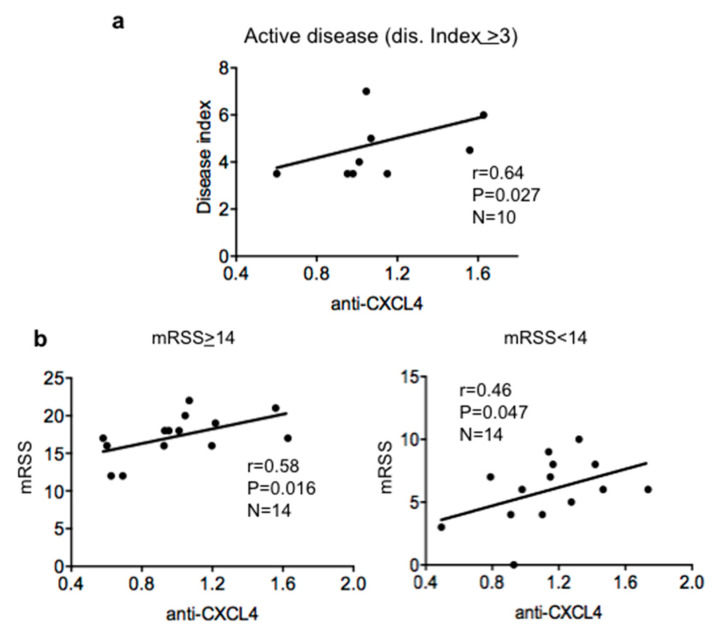
Anti-CXCL4 antibody reactivity can correlate with disease score (EScSGAI) and modified Rodnan skin score modified Rodnan skin score (mRSS) in active SSc. Sera of SSc1 (see Table S1) were tested for antibody reactivity to CXCL4 by ELISA as in Figure 1 and OD values obtained were correlated to the disease indexes (**a**) and mRSS (**b**) by Spearman correlation test. Only significant correlations are reported. Coefficient “*r*” of correlation, *p* values and sample size *n* are indicated on the graphs. In (**b**), we have divided the cohort of patients into high mRSS (>14) or low mRSS (<14).

By analyzing all patients of the discovery and replication cohorts, we did not find a significant correlation. However, when we concentrate the analysis on patients with more active disease (disease index >3), we could see a consistent correlation between the level of antibody response to CXCL4 and disease score, as measured by the European Scleroderma Study Group Activity Index (EScSGAI [17]) (Figure 2a). In the SSc patients with EScSGAI <3, we could not find a significant correlation. In the replication cohort, we found a moderate correlation between the intensity of anti-CXCL4 antibody reactivity and disease index in the group with less active disease (EScSGAI <3; Spearman’s *r* = 0.38, *p* = 0.05, *n* = 17). If the patients in the discovery cohorts were divided in two groups, those with modified Rodnan skin score (mRSS) <14 or mRSS >14, we found a positive and significant correlation between anti-CXCL4 antibody reactivity and mRSS, which was higher in the group of patients with mRSS >14. In the replication cohort, we found a positive correlation between the anti-CXCL4 antibody response and mRSS in patients with mRSS <14 (Spearman’s *r* = 0.4; *p* = 0.047, *n* = 17). SSc subtype (early or long lasting diffuse or limited subtypes) correlation analyses did not indicate any correlation with disease parameters such as erythrocyte sedimentation rate (ESR; values), C-reactive protein (CRP; negative/positive), creatinine kinase (CK; normal or altered), digital ulcers (DU; presence/absence), ACA (presence/absence) or ATA (presence/absence) (see Table S1), although this may be due to the low sample size of each single subtype. Overall, these results suggest that anti-CXCL4 antibodies could be markers of more severe and/or active disease. However, the results obtained with the two cohorts concord only partially with this assumption and data on SSc subtypes may need further analysis in larger SSc cohorts.

### 2.3. Anti-CXCL4 Antibody Reactivity in SSc Patients Correlates with Serum/Plasma IFN-α and Is Higher in SSc Patients with Lung Fibrosis

Since CXCL4 is a DNA/RNA binding protein, and knowing that circulating CXCL4–DNA complexes can activate pDCs to produce IFN-α [12] and possibly become the targets of anti-CXCL4 antibodies, we wondered whether the presence of anti-CXCL4 antibodies in SSc blood showed any relationship with blood IFN-α level.

**Figure 3:**
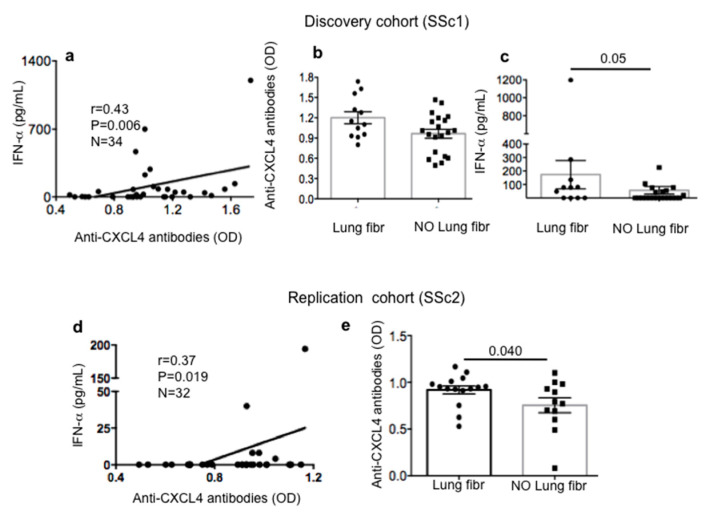
Anti-CXCL4 antibody reactivity correlates with circulating interferon (IFN)-I signature and is higher in patients with lung fibrosis. We measured IFN-α levels by ELISA in sera (discovery cohort) (**a**) or plasma (replication cohort) (**d**) of SSc patients and made a correlation with antibody response to CXCL4 tested by ELISA (OD). Correlation “*r*” was by Pearson’s test, significant *p* values and sample size *n* are indicated. Antibody reactivity to CXCL4 was compared between SSc patients with lung fibrosis (lung fibr) and patients with no lung fibrosis (NO lung fibr) (**b**,**e**) as well as circulating IFN-α levels (by ELISA) (**c**). In (**b**,**c**,**e**), horizontal bars are the means, vertical bars are standard error of the means, and *p* values are calculated by Mann–Whitney test.

We found that SSc1, our discovery cohort, showed a positive and significant correlation between autoantibody reactivity to CXCL4 and IFN-α levels, as detected by ELISA in sera (Figure 3a). Reactivity to CXCL4 was higher in lung fibrosis patients as compared to SSc patients with no sign of lung fibrosis, and the differences between the two groups were significant (Figure 3b). Thus, these data indicate that anti-CXCL4 antibody reactivity is not only an epiphenomenon, but could play a role in the IFN-I signature and fibrosis (two parameters that can be also related to each other) [7,8,12]. Indeed, in the discovery cohort, patients with lung fibrosis were those with higher serum IFN-α (Figure 3c). Most importantly, the correlation between serum IFN-α and anti-CXCL4 antibody reactivity was higher in SSc patients with lung fibrosis as compared to those with no lung fibrosis (Spearman’s *r* = 0.62, *p* = 0.016, *n* = 12 versus Spearman’s *r* = 0.37, *p* = 0.05, *n* = 20, respectively). We confirmed these results in the replication cohort (Figure 3d,e). SSc subtype analysis confirmed correlations between IFN-α and anti-CXCL4 antibody reactivity in the replication cohort (SSc2); of note in this cohort, as reported in Figure 1, anti-CXCL4 antibody reactivity was significant also in the early diffuse subgroup and not only in the diffuse long-lasting group. Remarkably, the correlation between the intensity of the anti-CXCL4 antibody response and IFN-α in plasma was present in the early diffuse group (*r* = 0.66, *p* = 0.03, *n* = 9) and in the long-lasting SSc group (Spearman’s *r* = 0.59, *p* = 0.037, *n* = 10). Lastly, it is of interest that anti-CXCL4 antibody reactivity in early limited SSc patients from the discovery cohort showed a consistent correlation with diffusing lung carbon monoxide (DLCO) by Spearman’s test (*r* = 0.75, *p* = 0.033, *n* = 7; see Table S1).

### 2.4. CXCL4 in Complex with Nucleic Acids Stimulates Memory B-Cells to Become Antibody-Secreting Plasma Cells

High and frequent antibody reactivity to CXCL4 could depend on the elevation of IFN-α in the circulation of SSc patients, as IFN-I is indeed boosting the production of antibodies in B-cells [18]. However, we wondered whether the adjuvant activity of CXCL4 in complex with nucleic acids could directly stimulate B-cells. To address this, we used an established protocol in which we isolated B-cells from HD PBMCs and stimulated them with CXCL4, DNA or CXCL4–DNA complexes for seven days [19,20]. As CXCL4 can form complexes with other polyanions, typically with heparin, we also used CXCL4 in complex with heparin to stimulate B-cells. In addition, we determined the effect of heparin on the CXCL4–DNA complex stimulation by treating the B-cells first with CXCL4–DNA complexes and adding heparin subsequently, or with CXCL4–heparin complexes with the addition of DNA afterwards. At the end of the culture, we estimated the percentage of B-cells that became CD19^neg^/CD27^high^/CD38^pos^/CD138^pos^ (namely plasma cells [19]). We also measured whether the differentiated cells secreted IgG in their supernatants at the end of the culture [19,20].

**Figure 4:**
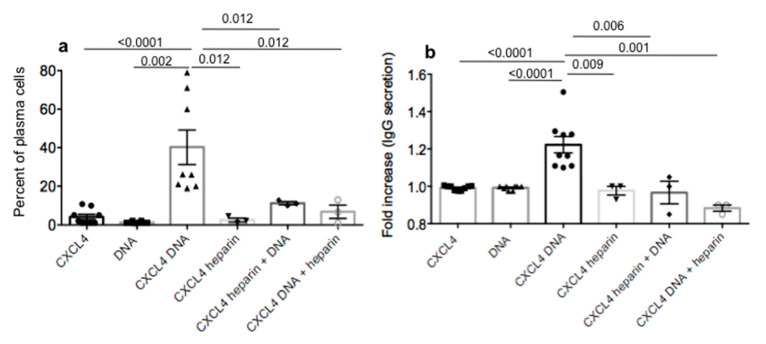
CXCL4–DNA complexes favor differentiation of antibody secreting plasma cells. B-cells isolated from HD were treated for 7 days as indicated (see Materials and Methods) and at the end of the culture, the percent of CD19^neg^CD27^high^CD38^pos^CD138^pos^ plasma cells was evaluated by flow cytometry (**a**). Supernatants were harvested at day 7 and analyzed for the concentration of IgG antibodies (**b**) by ELISA as outlined in the Methods section. IgG secretion is expressed as fold increase with respect to cells treated with CXCL4 alone. Horizontal bars are the means, vertical bars are the standard errors of the means, *p* values are by Mann–Whitney test (two-tailed). The results are from 2–4 independent experiments, made in duplicates/triplicates, and performed with purified B-cells from four different HD.

The results in Figure 4a show that the cultured B-cells were initially CD19^pos^/CD27^pos^ (namely memory B-cells) and CD38^low^, with CXCL4–DNA immune complexes able to induce a consistent proportion of these cells to differentiate into plasma cells in vitro after a seven-day culture. Secretion of total IgG tended to be significantly higher in the presence of CXCL4–DNA or –RNA (Figure S1) immune complexes (Figure 4b). Thus, the effect was likely mediated via TLR9, and possibly TLR7 stimulation [20,21,22]. Of note, CXCL4–heparin complexes did not exert any effect on B-cells, whereas heparin added after the challenge with CXCL4–DNA complexes, or heparin given in complex with CXCL4 followed by a subsequent treatment with DNA, abolished the stimulatory ability of CXCL4–DNA complexes. These results suggest that a further mechanism that can explain why anti-CXCL4 antibodies are high in SSc is that CXCL4 in complex with nucleic acids can act directly on B-cell maturation and the secretion of antibodies [20,21,22]. Of note, CXCL4–RNA complexes were also able to induce these effects, but only when the B-cells were pretreated at the beginning of the culture with IFN-α. These data suggest that the role of the autoantigen CXCL4 is two-fold: CXCL4–DNA/RNA complexes act as large particulate complexes (to stimulate antibody binding) and as an adjuvant for B-cells via their direct activation and indirectly by promoting IFN-α production by pDCs. Of note, the capacity of heparin, at least at the concentration used in the present experiments (5 UI/mL), to inhibit CXCL4–DNA complex stimulation suggests that heparin could be used in vivo to interfere with the immune amplification mediated by CXCL4 in complex with nucleic acids.

### 2.5. SSc T-Cells Proliferate to CXCL4 and Their Proliferation Correlates with Anti-CXCL4 Antibody Reactivity

The results above demonstrated that CXCL4–DNA complexes stimulate memory B-cells to become antibody-secreting plasma cells, in addition to amplifying the type I IFN signature, activating pDCs [12]. Both activities can implement anti-CXCL4 antibody reactivity. However, it is also known that the production of high affinity antibodies is ensured by CD4 T-cell help [23].

**Figure 5:**
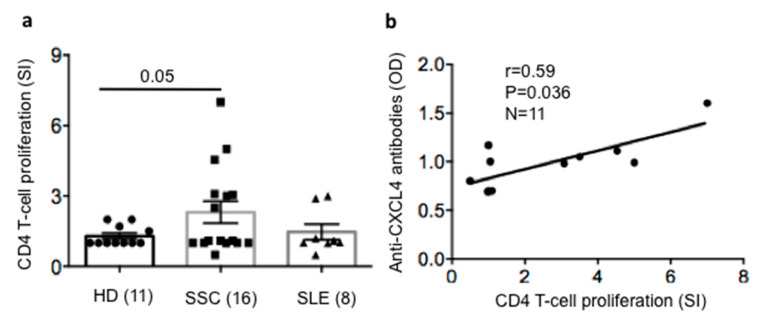
SSc-derived CD4 T-cells can proliferate in response to CXCL4 and their proliferation correlates with the level of anti-CXCL4 antibody reactivity. (**a**) PBMCs from SSc patients (SSC) or HD or SLE patients were either untreated or cultured for five days with 5 µg/mL of CXCL4, and then bromodeoxyuridine (BrdU) incorporation was evaluated in CD3^pos^CD4^pos^ cells. BrdU incorporation was assessed using an anti-BrdU antibody staining by flow cytometry (gating strategy in Figure S1). Results for proliferation are expressed as stimulation index (SI), as reported in the Methods. Horizontal bars are the means, vertical bars are the standard error of the means, *p* values by Mann–Whitney test (one-tailed). (**b**) Correlation between T-cell proliferation (reported as SI) and anti-CXCL4 antibody reactivity (OD), by Spearman’s correlation test. Spearman’s “*r*” coefficient, significance *p* and sample size *n* are reported.

Disposing of PBMCs from the SSc patients of the replication cohort, we could test the proliferative response of SSc T-helper cells to CXCL4 by bromodeoxyuridine (BrdU) incorporation, as previously done with other autoantigens [24,25]. As shown in Figure 5a, six out of 16 (38%) SSc patients had T-cells that proliferated to CXCL4 (the gating strategy for proliferation assays is reported in Figure S2). The available SLE patients were also tested, showing that CXCL4 can also be immunogenic in SLE. This was plausible, given the past determination of autoantibodies to CXCL4 in SLE which concords with our present data [16]. HD PBMCs responded poorly to CXCL4. Despite the limited number of SSc patients tested, CXCL4-specific T-cell responses in SSc were significantly higher than those obtained in HD. Remarkably, a positive and significant correlation between the presence of autoantibodies and T-cell proliferation to CXCL4 was apparent (Figure 5b).

Of note, we performed an accurate analysis using established algorithms with prediction servers (http://www.cbs.dtu.dk/services/NetMHCIIpan/) to forecast the likelihood of CXCL4 epitopes to bind human leucocyte antigen (HLA) -DR molecules, and therefore be presented to CD4 T-cells. We present the data of possible epitopes for the most common HLA-DR alleles expressed in Caucasians (DR4, DR1, and DR11 [26]) in Table S2, although other alleles (i.e., DR3, DR16, DR15 and DRB5, which are linked to SLE [27]), appeared able to bind more than one CXCL4-derived epitope (not shown).

These results indicate that CXCL4 has the ability to be recognized by T-cells in SSc; thus, we assumed that determinants of CXCL4 bind at least some MHC class II molecules. Overall, these data suggest that the generation of anti-CXCL4 antibodies may rely on T-cell help, as previously suggested in other contexts [14,16].

### 2.6. Autoantibodies to CXCL4 Can Implement IFN-α Production by pDCs

**Figure 6:**
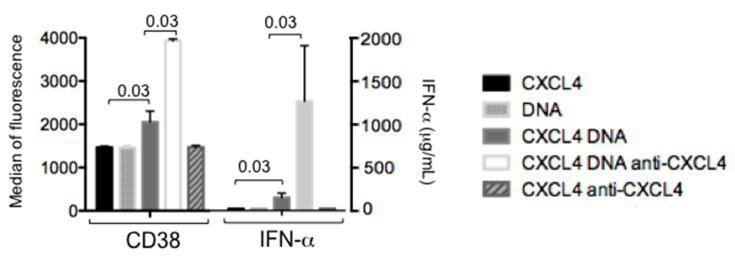
Complexes formed by CXCL4–DNA–anti-CXCL4 antibodies are more effective than CXCL4–DNA complexes at stimulating pDCs. Purified pDCs were treated as indicated (huDNA at 5µg/mL) and their expression of CD38 (IFN-induced gene), as well as IFN-α release, were detected by flow cytometry and ELISA, respectively. *p* values by Wilcoxon’s test are reported. Results are cumulative data of two independent experiments performed in triplicate.

We have assumed that that the adjuvant activity of CXCL4–nucleic acid immune complexes implements IFN-I production, which in turn boosts the memory B-cell capacity to produce antibodies to CXCL4. One possibility is that autoantibodies to CXCL4 directly increment IFN-I production, which is already ensured by the formation and activity of CXCL4–DNA/RNA immune complexes [3,12]. To understand this, we prepared a CXCL4–DNA–IgG immune complex by pre-mixing CXCL4–DNA and an anti-CXCL4 antibody (see Methods) and challenged purified pDCs with this mixture. As a control, the same antibody was given to pDCs stimulated by CXCL4 alone. Figure 6 shows that a suboptimal stimulation of pDCs by CXCL4–huDNA complexes can induce a certain IFN-I response (seen as CD38 u-regulation, as CD38 is an IFN-induced factor [28,29]) and detection of low level IFN-α, as measured by ELISA. However, this response can be greatly augmented by the presence of anti-CXCL4 antibodies in the stimulatory complex. These results, although obtained with an artificial anti-CXCL4–CXCL4–DNA complex, suggest that one of the effector functions of anti-CXCL4 antibodies could be to concentrate and/or more efficiently deliver CXCL4–DNA complexes to immune cells (i.e., to pDCs) and further amplify IFN-α production in SSc. This finding also suggests the interesting possibility that CXCL4–anti-CXCL4 immune complexes circulating in SLE (or in other disease) could be interferogenic because they contain nucleic acids [14,16].

## 3. Discussion

In this study, we have shown that CXCL4, a molecule with pleiotropic functions on immune and non-immune cells [30] and a biomarker in SSc [7], represents a new autoantigen in SSc. Both antibodies and T-cells seem to react to CXCL4 in SSc. These antibodies do not correlate with specific clinical or inflammatory parameters, except DLCO in one cohort. However, our analysis shows that they are significantly higher in SSc patients with active disease. The results of the two cohorts are not completely in agreement. This could be due to the difference inherent in the two cohorts, where SSc1 includes patients with a higher disease score (EScSGAI) than SSc2 (SSc1: EScSGAI range 1–8, mean = 2.77; SSc2: EScSGAI range 1–4.5, mean: 1.9). However, larger cohorts could confirm these data.

Interestingly, anti-CXCL4 antibodies appear to correlate with blood IFN-α and are higher in pulmonary fibrosis in both SSc cohorts examined. This is of interest, as here we show that anti-CXCL4–huDNA–IgG immune complexes can indeed induce pDC-mediated immune amplification via implementation of the IFN-α secretion already mediated by CXCL4–huDNAcomplexes [12]. Thus, anti-CXCL4 antibodies may act to increment the SSc IFN-I signature in vivo. It has been shown that IFN-I can be deleterious in SSc for several reasons (reviewed elsewhere [3,11,31]) and, if present early during the disease course, can be dangerous and predict a worse prognosis and lung fibrosis [11]. It is interesting that in our discovery cohort, patients with pulmonary fibrosis are not only the ones with higher anti-CXCL4 antibody reactivity, but also those with a higher IFN-I signature in the blood.

The capacity of anti-CXCL4 antibodies to increase the effect of CXCL4–huDNA complexes on pDC activation and IFN-α release can be mediated by facilitation of CXCL4–DNA complex entry into the pDCs via Fc receptors. This phenomenon has been described for anti-LL37 antibodies, where LL37 is a molecule endowed with adjuvant effects on IFN-I secretion similar to those mediated by CXCL4 [32]. In keeping with this, we have demonstrated previously that an anti-Fc-gamma receptor-blocking antibody could, at least in part, inhibit the effect of DNA-treated SSc plasma (positive for CXCL4) on pDC activation [12].

Our study does not clarify the timing of anti-CXCL4 generation. In the discovery cohorts, anti-CXCL4 antibodies correlate with disease duration, indicating that some time is required to generate anti-CXCL4 antibody reactivity from SSc onset. However, in the replication cohort, anti-CXCL4 antibodies were detectable in early patients as well, suggesting that these antibodies can be present at early stages. Studies are required to understand whether anti-CXCL4 antibody reactivity can be present even before disease onset (such for ATA and ACA [1,2,3,4]). We plan to study a cohort of patients classified according to “very early diagnosis of systemic sclerosis” (VEDOSS) to illuminate this aspect [33].

Our view is that frequent anti-CXCL4 antibody generation in SSc is particularly favored by the fact that the antigen, CXCL4, is endowed with adjuvant activity. Indeed, we show that B-cells themselves can be stimulated by CXCL4–DNA and even CXCL4–RNA complexes to become antibody-secreting plasma cells. This effect is obtained in in vitro assays and is likely to act on memory B-cells after seven days of culture in the presence of CXCL4–DNA complexes. To elucidate the effect on naïve B cells, further experiments should be performed on purified naïve B-cells, possibly also derived from SSc patients. The possibility that T-cells are necessary for the generation of high affinity anti-CXCL4 antibodies in SSc via T-cell derived cytokines and receptor–co-receptor binding is highly likely [23]. Indeed, although preliminary, our results reveal that T-cells do proliferate to CXCL4 and this proliferative capacity correlates in a significant manner with antibody reactivity to CXCL4. The next step will be to study the polarization of these T-cells in terms of cytokine production and surface markers. We anticipate that in some patients, CXCL4-proliferating cells are CCR10 positive, suggesting they could home to the skin [4,34,35].

Anti-CXCL4 antibodies have also been described in SLE and were shown to correlate with disease activity (SLEDAI [16]). In SLE, both heparin-dependent and heparin-independent antibodies have been detected but only the heparin-independent antibodies were found to correlate with SLEDAI [16]. Our ELISA is made using CXCL4 as the antigen; thus, we assume that we are looking at heparin-independent anti-CXCL4 antibodies in our SSc-cohorts. Of course, we cannot completely exclude that sera/plasma components bind to CXCL4 coated on the ELISA plates during the assays.

It is worth noting that a rare disease called heparin-induced thrombocytopenia (HIT), in which CXCL4 becomes the target of anti-CXCL4 antibodies with pathogenic activity, is a severe condition in which heparin-dependent anti-CXCL4 antibodies are generated that exert pathogenic functions as they target CXCL4 attached to endogenous heparan-sulfate on endothelial cells, causing injury to these cells [14]. HIT antibodies can also bind independently of heparin to CXCL4–proteoglycan (chrondroitin sulfate) complexes and also cause normal platelets to aggregate and activate [14]. Of note, platelet activation is present in SSc [36].

We are also currently testing whether heparin-dependent antibodies are present in SSc. Indeed, these antibodies can be found even in patients that never received exogenous heparin to treat thrombotic conditions [14]. The requirement for CXCL4–heparin to assemble into ultra-large antigenic complexes (ULCs) is likely important for initiating immune responses, as confirmed in mouse models. However, binding of CXCL4 to other polyanions, such as cell surface glycosaminoglycans (GAGs), [37,38] and even DNA [39,40] has been proposed to explain so-called “spontaneous” HIT in patients with no prior heparin exposure [14,41]. Given the circulation of CXCL4–DNA complexes in SSc blood and their presence in SSc skin, we believe that CXCL4 becomes immunogenic due to its binding to anionic DNA or RNA in SSc [12].

Our results on CD4 T-cell proliferation to CXCL4 are relevant also for HIT [14]. It has been hypothesized that the presence of high-titer antigen-specific IgG responses to CXCL4 in HIT denotes the involvement of adaptive immunity, and highlights a role for T-helper cells in the generation of these antibodies [14,42]. A restricted T-cell repertoire in patients with HIT [14,41,43] has been noted. However, despite this evidence, T-cells proliferating to CXCL4 have not been studied. Thus, our proliferation assays show that T-cells can recognize epitopes within CXCL4. Moreover, the use of a specific algorithm to predict the binding capacity of CXCL4-derived epitopes to MHC, and therefore the potential immunogenicity for CD4 T-cells, indicates the presence of “binding motifs” for the most diffuse HLA-DR alleles (DR4, DR1, and DR11) in Caucasians, and for possibly other alleles as well.

In conclusion, our study reports a novelty in SSc: anti-CXCL4 antibody reactivity is frequently generated and accompanied by CXCL4-induced T-cell proliferation, which indicates that CXCL4 is acting as a novel autoantigen in SSc. Larger studies are certainly required to reproduce these findings. However, our study also indicates that anti-CXCL4 antibodies could contribute to the overall IFN-I signature in SSc. The results reinforce the assumption that CXCL4 can bind polyanionic compounds to become immunogenic [12,14]). It is not clear whether anti-CXCL4 antibodies can also bind CXCL4 on endothelial cells in SSc, causing problems in blood vessels. The activation of platelets, which is present in SSc, can be also determined by anti-CXCL4 antibodies, whereas T-cells specific for CXCL4 could migrate to CXCL4-expressing tissues and cause inflammation.

We believe that the present results in SSc have attractive implications for SSc and for diseases other than SSc (i.e., HIT). Indeed, they may stimulate future directions of research in SSc and suggest the intriguing possibility that the supposed adjuvant activity of CXCL4–heparin immune complexes may rely on cell-free DNA, which is possibly contained in these complexes.

The limitations of this study reside in the fact that the phenotype of SSc T-cells specific for CXCL4 has not be studied and therefore the T-cell help for antibody production is guessed on the basis of CD4 T-cell proliferation. In addition, the possible presence of immunodominant T-cell epitopes within the CXCL4 sequence is hypothesized but not experimentally demonstrated and relies on the specific prediction server mentioned in the Methods section and in Table S2. Finally, although anti-CXCL4 antibody reactivity was shown against CXCL4 alone, this does not exclude that some of the antibodies identified in the SSc patients are heparin-dependent. Future studies, in larger cohorts, may address these issues.

## 4. Materials and Methods

### 4.1. Human Study and Samples

SSC blood (20 mL), and blood from UC patients were obtained in Rome, Italy, Policlinico Umberto I, Department of Internal Medicine and Medical Specialties—Rheumatology Unit or University Hospital of Geneva, CH, and Sandro Pertini Hospital, Rome, respectively. SLE samples were from the Swiss SLE cohort study (SSCS); PBMCs and plasma or sera from HD, matched for age and sex with SSc as much as possible, were from blood centers in Policlinico Umberto I, Italy and Geneva University Hospital, Switzerland. SSc patients satisfied the American college of rheumatology (ACR)/European League Against Rheumatism (EULAR) 2013 classification criteria. Disease activity was measured by EScSGAI [17]. Disease activity in SLE patients was assessed by SLEDAI 2000 [44]. UC disease activity was assessed by endoscopic Mayo 58 and UC patients with clinical and endoscopic activity (Mayo Score Full ≥3) were evaluated. Blood samples were collected at the time of endoscopy [45]. To corroborate data, we used two different SSc cohorts: for the discovery cohorts, we could collect sera, but for the replication cohort, we collected both PBMCs (in part of the patients) and plasma. Exclusion criteria included patients treated with biologics.

All samples were obtained upon approval by Ethic Committees of University La Sapienza or UNIGE (University Hospital) (rif.1725, rif.2125, IT; 2017-01434, Geneva); Sandro Pertini Hospital in Rome, and Swiss Ethics, Switzerland. All blood donors gave informed consent according to the Helsinki declaration.

### 4.2. Antigens, Antibodies for Functional Assays and Flow Cytometry

Human recombinant CXCL4 was obtained from Sino Biological (Beijing, China) and we also used CXCL4 synthesized by Biomatik (Ontario, Canada), as reported [12].

Antibodies to CXCL4 for pDC stimulation were from Biotechne (Minneapolis, MN, USA, human CXCL4/PF4 Antibody antigen affinity-purified Polyclonal Goat IgG, Catalog Number: AF795). Antibodies to CD4, CD8 and CD3, were conjugated with various fluorochromes: fluorescein isothiocyanate, (FITC), phycoerythrin (PE), peridinin-chlorophyll-protein (PerCp), allophycocyanin (APC), phycoerythrin-cyanine7 (PE-Cy7), AlexaFluor488, and were from BD Biosciences or eBiosciences (San Diego, CA, USA). For cell characterization, FITC-, APC-, PE-, PerCp-, PerCpCy5.5-, APC-Cy7-, AlexaFluor488-conjugated CD38, CD138, CD19, CD20, CD27, antibodies and relative isotype control antibodies, were purchased from BD Biosciences, eBiosciences, Novus Biologicals (Littleton, CO, USA), R&D (Minneapolis, MN, USA) and Life Technologies (Monza MB, Italy).

### 4.3. T-Cell Proliferation Assay

Patients/HD PBMCs were purified from Ethylenediaminetetraacetic acid (EDTA)-treated blood on Ficoll-Hypaque (Pharmacia Fine Chemicals, Uppsala, Sweden) and were incubated (1 × 10^5^ cells/well) in 96-well-flat-bottom-microplates (BD) in T cell-medium (RPMI 1640, Thermofisher, 10% heat-inactivated human serum (HS), Gibco, Thermo Fisher, Waltham, MA, USA), 2 mM L-glutamine, 10 U/mL penicillin, and 100 µg/mL streptomycin), with/without peptides. We performed assays on fresh cells, within 1–3 h from collection, or on PBMCs frozen in 90% Fetal calf serum (FCS) + 10% dimethyl sulfoxide (DMSO, Sigmaaldrich, MO, USA), whose viability was assessed by Trypan blue exclusion on an inverted microscope. Recovery of live cells was between 65% and 85% of the frozen number. At days 3 and 5, BrdU was added (1 µg/mL). BrdU incorporation was detected by APC-labeled anti-BrdU antibody (BD Pharmingen, San Jose, CA, USA), after surface staining for CD4/CD3/CD8, by flow cytometry. The internal control for T-cell viability and proliferation was usually phytohemagglutinin (PHA) treatment (2 µg/mL) or tetanus toxoid (TT) treament (1 µg/mL). SI for proliferation was calculated by dividing the percent of BrdU staining in the presence of the peptide used to stimulate cells by the percent of BrdU staining in the absence of peptide stimulation. SI for proliferation was considered positive when >3. This cut-off was obtained by calculating the mean ±2 (standard deviations, DS) (1.5 ± 2 (0.6)) (of the stimulation indexes calculated as above) of the proliferation of HD. The basal percentage of proliferation was set using an isotype matched antibody (APC-isotype control) as a control and never exceeded 5% of the cell proliferation value (background proliferation) [12]. Assays were repeated twice with each patient. In rare cases in which background proliferation exceed antigen-specific proliferation, the assay was repeated. Concomitant phenotype analysis of CXCL4 responder T-cells could include staining for CXCR5 and CCR10 [24,25,35,36].

Cells were analyzed on a FACS Gallios, and analyzed by Kaluza or by Flowjo (Tristar, USA).

### 4.4. Production of Human/Bacterial DNA/RNA

HuDNA or bacDNA preparations were purchased or extracted from PBMCs or *Escherichia coli* (*E. Coli*) cultures, respectively (as reported [12]) and were fragmented by sonication. Five milligrams of DNA in a volume of 300 µL were fragmented using the Sonics Vibra Cell sonicator (Sonics & Materials Inc., Newtown, CT, USA), with the following settings: 2, 4, and 10 sonication cycles (30 s on, 30 s off in ice) to obtain DNA fragment sizes between 100 and 1000 bp. The resulting size distribution was controlled by 2% agarose gel electrophoresis.

This digestion was done to mimic the degradation of DNA in an extracellular environment in vivo. Human RNA was extracted from PBMCs and bacterial RNA from *E. coli* cultures.

### 4.5. Isolation and Stimulation of pDCs

Buffy coats were from Centro Trasfusionale, Policlinico Umberto I, Rome, IT. After separation of mononuclear cells by Ficoll (GE Healthcare, Westborough, MA, USA) centrifugation, pDCs were purified as described [12] using the Diamond Plasmacytoid Dendritic Cell Isolation Kit (Miltenyi Biotec, Bergisch Gladbach, Germany) to obtain 99% purity.

pDCs were stimulated with CXCL4 (1 µM) and huDNA (5µg/mL) that were premixed for 20 min at room temperature before stimulating the pDCs. Anti-CXCL4 (5 µg/mL) antibody was added directly to CXCL4 and then huDNA was added.

### 4.6. IFN-α Determination in pDC Cell Cultures and Sera/Plasma

IFN-α was determined by ELISA (MabTech, Cincinnati, OH, USA) as described [12]. Sera and plasma were diluted 1:4 in phosphate buffer solution (PBS); culture supernatants were diluted from 1:4 to 1:10 depending on the stimulus used.

### 4.7. Isolation of B-Cells from Buffy Coats

Human peripheral blood mononuclear cells (PBMCs) were isolated by Ficoll-Paque centrifugation (GE Healthcare, MA, USA). Total B-cells were isolated by using the Human B-Cell Isolation Kit II (Miltenyi Biotec, Bergisch Gladbach, Germany), from fresh PBMCs. The purity of B-cells was verified by flow cytometry using monoclonal anti-human CD19 fluorescein isothiocyanate (FITC). The percentage of memory B-cells was assessed by flow cytometry using monoclonal anti-human CD19 fluorescein, anti-human CD27 allophycocyanin and anti-human CD38 Pacific Blue antibodies (BD Biosciences, San Jose, CA, USA). The majority of B-cells were memory cells expressing CD19/CD27 and low CD38 [19,20].

### 4.8. Stimulation of Purified B-Cells

CXCL4 was premixed with DNA (10 µg/mL) or RNA (15 µg/mL) and added to the B-cell cultures after a 15-min incubation at room temperature. The concentration of CXCL4 was between 1–2 µM. Complexes were formed with huDNA/BacDNA, or huRNA/BacRNA. In some experiments, B-cells were stimulated with CXCL4 (1 µM) in complex with low molecular weight heparin (calcium nadroparin, 5 UI/mL). Moreover, cells were treated with CXCL4–DNA complexes, followed by treatment, after 30 min with heparin at 5 UI/mL; alternatively, cells were treated with CXCL4 (1 µM) plus heparin (5 UI/mL) and, after 30 min, DNA (10 µg/mL) was added to the cultures.

Maturation of B-cells into plasma cells was investigated by flow cytometry (Gallios, Beckman Coulter, Brea, CA, USA) with monoclonal anti-human CD19 fluorescein, CD27 allophycocyanin, CD38 Pacific Blue, CD138 phycoerythrin (BD Biosciences, CA, USA) antibodies at day 7.

### 4.9. ELISA for Anti-CXCL4 Autoantibodies and Human IgG Detection in Sera/Plasma and Culture Supernatants, Respectively

Anti-CXCL4 antibodies were measured by ELISA using a modified method already described in [20,25,32]. Briefly, 96-well flat-bottom plates (non-binding surface polystyrene, Corning, Corning, NY, USA) were coated with 2 µg/mL CXCL4 in carbonate buffer (0.1 M NaHCHO_3_, pH 9) for 2 h (or overnight) and washed four times with PBS + 0.1% Tween-20. This washing buffer was used for washing at all steps. Blocking buffer containing 2% bovine serum albumin (BSA, Sigma-Aldrich, St. Louis, MO, USA) in PBS was used for at least 1 h (or overnight) to saturate unspecific binding sites. After washing, sera or plasma were diluted at various concentrations (usually 1:100 or 1:200) in PBS + 2% BSA, followed by a 1 h incubation with a horseradish peroxidase (HRP)-conjugated goat anti-human IgG (Sigma-Aldrich, St. Louis, MO, USA) diluted 1:5000 in PBS. The color was developed for 5 min with 3,3′,5,5′-tetramethylbenzidine (TMB) substrate (Sigma-Aldrich). The reaction was stopped by adding 50 µL of 2 N H_2_SO_4_, and absorbance determined at 450 nm with a reference wavelength of 540 nm. Anti-CXCL4 were considered positive and significant when they exceed the mean OD values obtained with HD, plus two standard deviations (SD).

Anti-human IgG antibody (Sigma-Aldrich, St. Louis, MO, USA) for assessing human IgG in the supernatants of B-cells was diluted 1:100 in carbonate buffer (0.1 M NaHCHO_3_, pH 9) for 2 h (or overnight) and cell culture supernatants were diluted 1:2 or 1:4 [20].

### 4.10. Statistical Analyses

Differences between mean values were assessed by Wilcoxon’s matched-pairs signed rank test, or Mann–Whitney test (one tailed or two tailed), especially in cases of low patient sample sizes. Statistical significance was set at *p* < 0.05. Correlation analyses were performed by Pearson’s or Spearman’s rank correlation tests, depending on the sample size (*n*). For low sample sizes, we always used Spearman’s correlation. Data were analyzed and correlations were performed using GraphPad Prism 7.0.

## 5. Conclusions

This paper found that: (1) CXCL4 is a new autoantigen for both B-cells and T-cells in SSc; (2) antibodies to CXCL4 correlate with the IFN-I signature; and (3) anti-CXCL4 antibodies implement IFN-α induced by CXCL4–DNA complexes in pDCs via TLR9.

The study also shows for the first time that CXCL4–DNA/RNA complexes can directly stimulate B-cells to mature into antibody secreting plasma cells and that this transition may be abolished by heparin, although further work is required to confirm this finding.

Thus, in a hypothetical model, CXCL4–DNA/RNA complexes stimulate pDCs, IFN-I and B-cells, to boost autoantibody production, including antibodies to CXCL4. The latter can bind CXCL4–DNA complexes that circulate in SSc and further increase their capacity to stimulate pDCs and IFN-α release. This harmful loop can be operative in SSc and possibly in other pathological conditions in which high CXCL4 levels are reached in the body, and cell-free DNA/RNA is copiously released following injury, inflammation or as the consequence of different forms of cell death.

## Supplementary Materials

Supplementary materials can be found at https://www.mdpi.com/1422-0067/21/14/5102/s1.
