# Supplementary material for: Anti-CXCL4 Antibody Reactivity Is Present in Systemic Sclerosis (SSc) and Correlates with the SSc Type I Interferon Signature
Source: Int J Mol Sci. 2020 Jul 19;21(14):5102. doi: 10.3390/ijms21145102 (PMC7404208; doi:10.3390/ijms21145102)
Supplement: Supplementary file 1 [file ijms-21-05102-s001.pdf]

Supplementary Materials

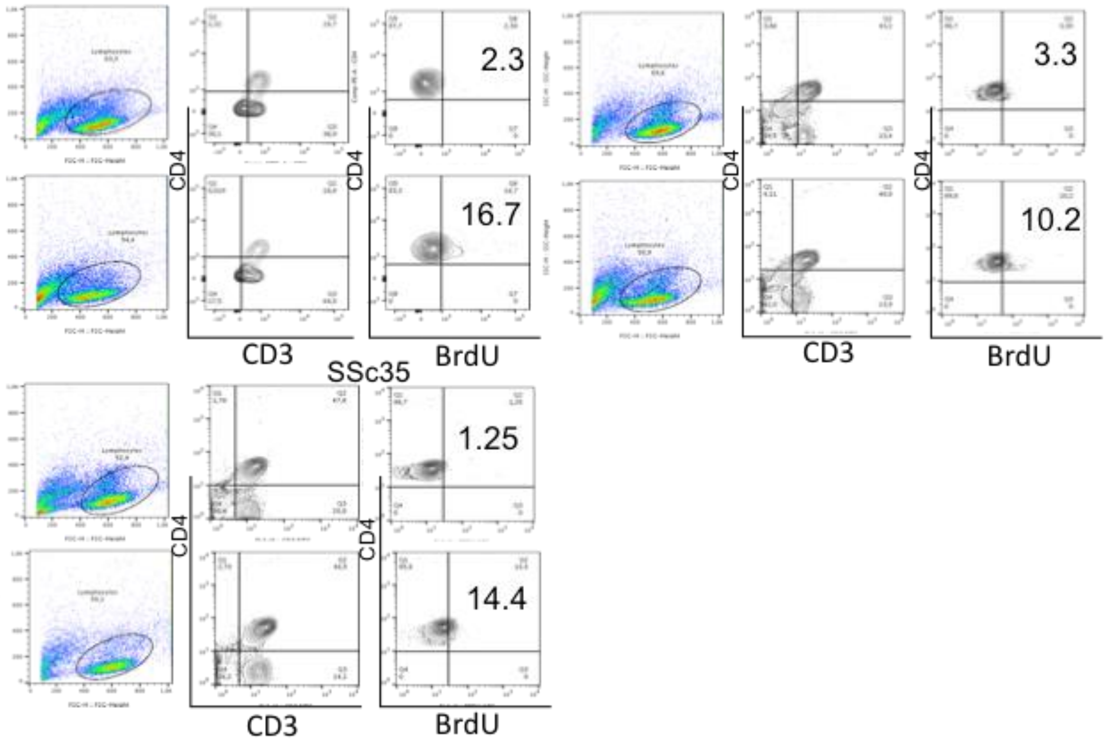

Figure S1. Gating strategy for T-cell proliferation assays. PBMCs were cultured for 5 days In the presence of CXCL4 (5 mg/mL) or were untreated. Percent of proliferation (% of BrdU incorporation, BrdU) is shown on the gated CD4+CD3+ cells in the T-lymphocytes gate. Percent of BrdU incorporation is reported on the contour plots on the right. Cells were analyzed by flow cytometry.

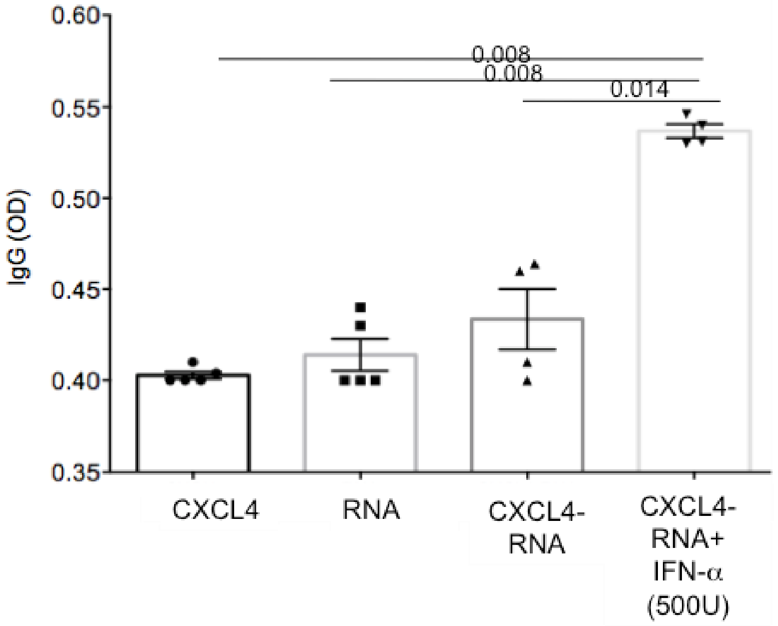

Figure S2. Treatment of B-cells with CXCL4-RNA complexes and IFN-a significantly increases antibody release in supernatants of B-cells. B-cells were treated as indicated for 7 days and concentration of antibodies (IgG) was tested by ELISA. Results reported as OD of different experiments. Horizontal bars are the means, vertical bars standard error of the means, *p* values by Mann-Whitney test.

Table S1. Clinical characteristics of the studied cohorts.

Patients	SSc1 (35)	SSc2 (32)	SLE1 (19)	SLE2 (16)	UC (48)	HD (50)
Age, mean (range) years	58 (28–71)	53 (39-61)	47 (25–70)	48 (34-69)	48 (20-77)	50 (26–58)
Sex (M/F):	2/33	1/30	2/17	1/16	35/23	11/19
Disease duration with range:						
Months	99.6 (12–600)	135 (1-455)	48 (12–500)	40 (12–425)	144 (0-432)	N/A
SLEDAI	N/A	N/A	9 (0-18)	N/A	N/A	N/A
Form (limited/diffuse)	14/21	5/27	N/A	N/A	N/A	N/A
Early limited/early diffuse	9/7	3/10	N/A	N/A	N/A	N/A
EScSGAI	2.77 (1-8)	1.9 (1-4.5)	N/A	N/A	N/A	N/A
mRSS (mean, range)	11.6 (0–22)	13.1 (0-36)	N/A	N/A	N/A	N/A
ESR (mean, range)	24.9 (2-75)	22.3 (2-64)	N/A	N/A	N/A	N/A
Other clinical and laboratory characteristics (%):						
ANA positivity	94%	84%	N/A	N/A	N/A	N/A
ACA positivity	57%	21%	N/A	N/A	N/A	N/A
ATA positivity	31%	62%	N/A	N/A	N/A	N/A
aRNAP3 positivity	6%	3%	N/A	N/A	N/A	N/A
CRP positive	20%	12%	N/A	N/A	N/A	N/A
ERS >30	23%	25%	N/A	N/A	N/A	N/A
Raynaud Phenomenon	100%	100%	N/A	N/A	N/A	N/A
DU	56%	60%	N/A	N/A	N/A	N/A
Teleangectasia	N/A	50%	N/A	N/A	N/A	N/A
Pulmonary Arterial Hypertension	14%	N/A	N/A	N/A	N/A	N/A
Lung fibrosis (yes/no)	12/20	15/12	N/A	N/A	N/A	N/A
DLCO (%) (mean)	70%	54%	N/A	N/A	N/A	N/A
DLCO <80%	54%	66%	N/A	N/A	N/A	N/A
Gastrointestinal involvement	51%	59%	N/A	N/A	N/A	N/A
Synovitis	33%	50%	N/A	N/A	N/A	N/A
Tendon friction rubs	40%	35%	N/A	N/A	N/A	N/A
CK elevation	34%	6%	N/A	N/A	N/A	N/A
DMARDs	80%	80%	60%	-	-	N/A

DU and synovitis were clinically defined, disease duration refers to the time from the onset of the first non-Raynaud's phenomenon manifestation. ACA, anti-centromere antibody; ANA, anti-nuclear antibodies; ATA, anti-topoisomerase-I antibody; aRNAP3, anti-RNA polymerase III; CK, creatine kinase; DU, digital ulcer; ILD, interstitial lung disease, defined by high resolution computed tomography; DMARDs, disease modifying antirheumatic drugs; mRSS, modified Rodnan skin score; N/A, not applicable; SSc, systemic sclerosis; HD healthy donors, SLE, systemic lupus erythematosus; UC: ulcerative colitis patients; ESR, erythrocyte sedimentation rate; CRP, c-reactive protein; DLCO, diffusion lung CO.

Table S2. CXCL4 protein contains sequences that bind the most diffuse HLA-DR alleles in Caucasians and can be potentially presented to T helper cells.

Potential Immunogenic Epitopes in Cxcl4 Sequence.		
**aa Pos.**	**Alleles**	**15aa Sequence**
19	DRB1_0401 **VRPRHITSLEVIKAG**	WB
20	DRB1_0401 **RPRHITSLEVIKAGP**	SB
21	DRB1_0401 **PRHITSLEVIKAGPH**	SB
22	DRB1_0401 **RHITSLEVIKAGPHC**	WB
19	DRB1_0101 **VRPRHITSLEVIKAG**	WB
20	DRB1_0101 **RPRHITSLEVIKAGP**	SB
21	DRB1_0101 **PRHITSLEVIKAGPH**	SB
22	DRB1_0101 **RHITSLEVIKAGPHC**	WB
19	DRB1_1101 **VRPRHITSLEVIKAG**	WB
20	DRB1_1101 **RPRHITSLEVIKAGP**	WB
21	DRB1_1101 **PRHITSLEVIKAGPH**	WB
22	DRB1_1101 **RHITSLEVIKAGPHC**	WB
36	DRB1_1101 **CPTAQLIATLKNGRK**	WB
37	DRB1_1101 **PTAQLIATLKNGRKI**	WB
55	DRB1_1101 **LQALLYKKIIKEHLE**	WB
56	DRB1_1101 **QALLYKKIIKEHLES**	WB
55	DRB1_1101 **LQALLYKKIIKEHLE**	WB
56	DRB1_1101 **QALLYKKIIKEHLES**	WB

The server http://www.cbs.dtu.dk/services/NetMHCIIpan/ estimated the possible 15 amino acid (aa) long over-lapping peptide sequences, contained in CXCL4, which bind the most common HLA-ClassII alleles DRB1*0401, DRB1*0101, DRB1*1101. WB = week binder, SB = strong binder.
